# Supplementary material for: Structure and mechanism of the type I-G CRISPR effector
Source: Nucleic Acids Res. 2022 Oct 28;50(19):11214–28. doi: 10.1093/nar/gkac925 (PMC9638904; doi:10.1093/nar/gkac925)
Supplement: gkac925_Supplemental_Files [file gkac925_supplemental_files.zip › Revised supplementary data.pdf]

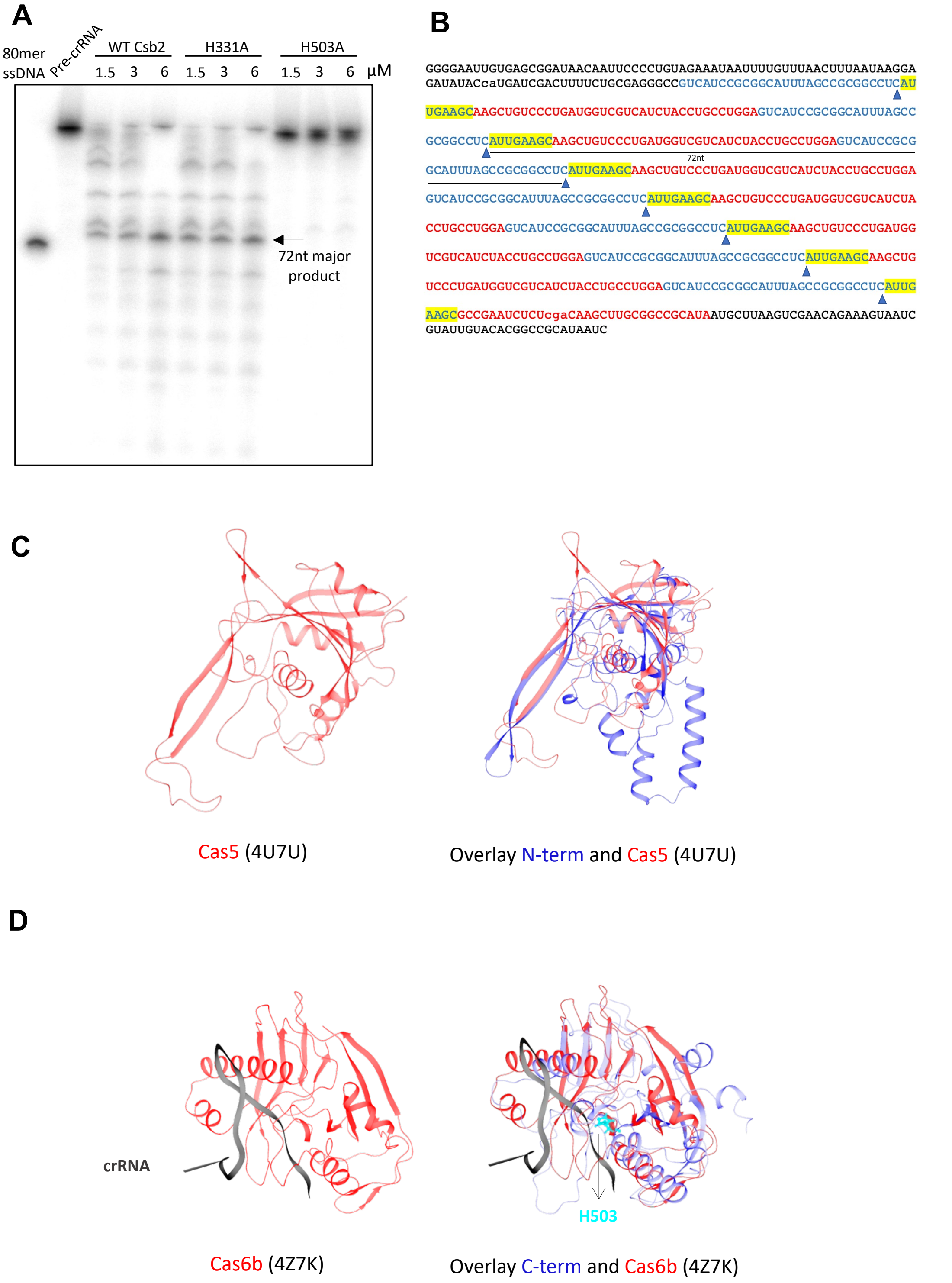

Figure S1. pre-crRNA cleavage by Csb2 (A) In vitro transcribed 643 nt pre-crRNA was cleaved by WT-Csb2 or mutant of Csb2. **(B)** The sequence of this 643 nt pre-crRNA, arrow indicates the cleavage site. **(C)** Structural comparison of the modelled Csb2 N-terminal domain (blue) with the Cas5 protein from *Escherichia coli* (red). **(D)** Structural comparison of the modelled Csb2 C-terminal domain (blue) with the Cas6b protein from *Methanococcus maripaludis* (red) reveals the likely site of crRNA hairpin binding (black, from the Cas6b structure) adjacent to the position of the H503 (cyan) active site residue.

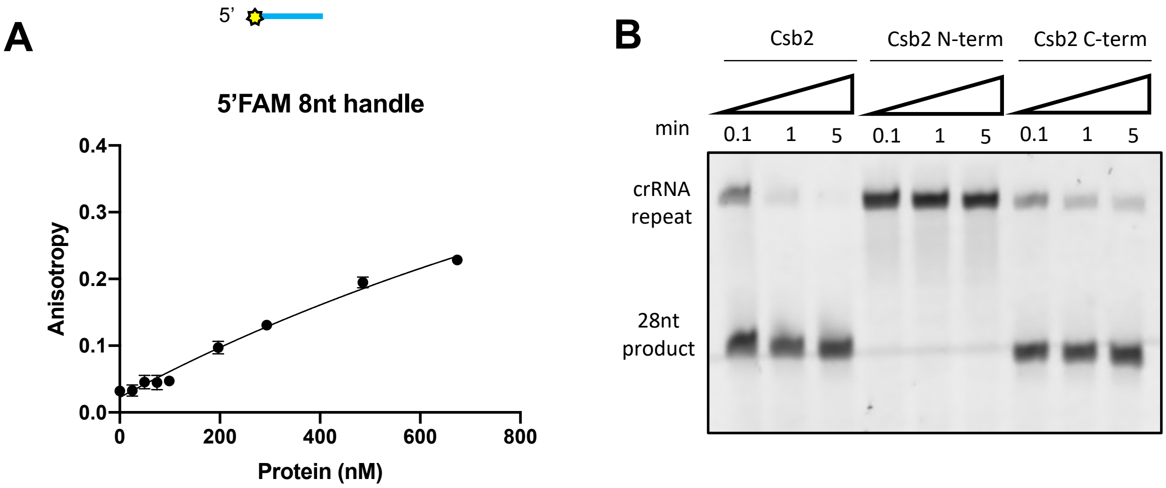

Figure S2. Properties of the isolated Csb2 N- and C-terminal (A) Anisotropy showing Csb2 binding affinity with 5'-6FAM-labelled 8nt handle. Data points and error bars represent the mean of five technical replicates and standard deviation. **(B)** The N-terminal domain of Csb2 does not cleave the repeat, but C-terminal domain alone does.

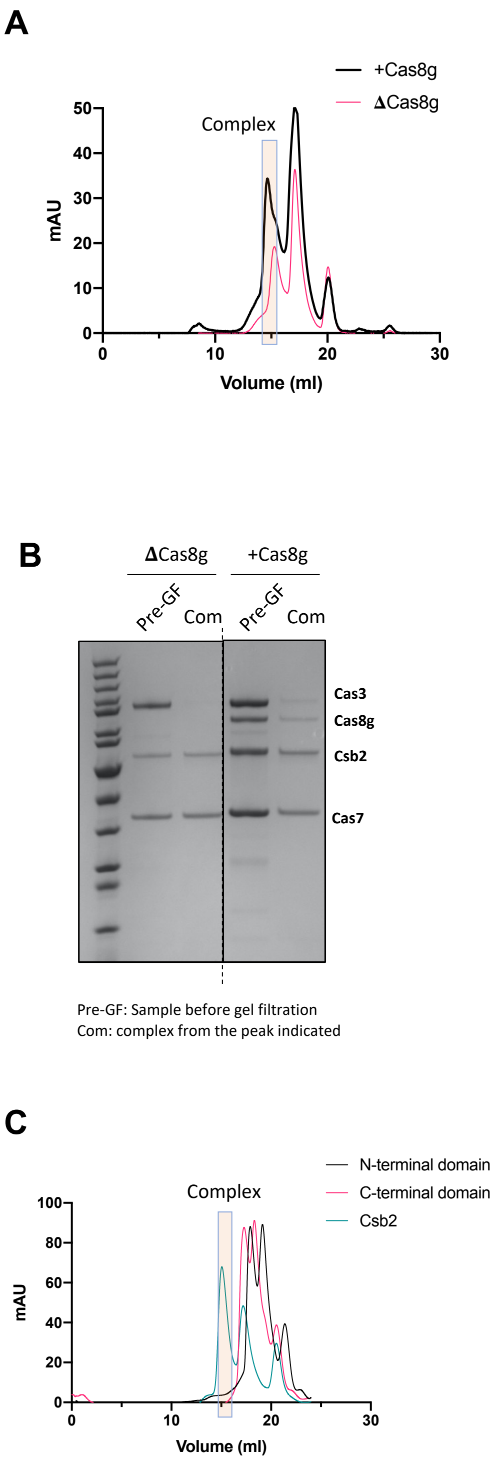

Figure S3. Effector complex formation. (A) Chromatography showing complex formation in the presence of Cas8g or in the absence of Cas8g. The rectangle indicated the fraction of the complex. **(B)** The complex from chromatography was submitted to SDS-PAGE electrophoresis. **(C)** Chromatography showing only intact csb2 formed the effector complex, neither N-terminal domain nor C-terminal domain alone formed the complex.

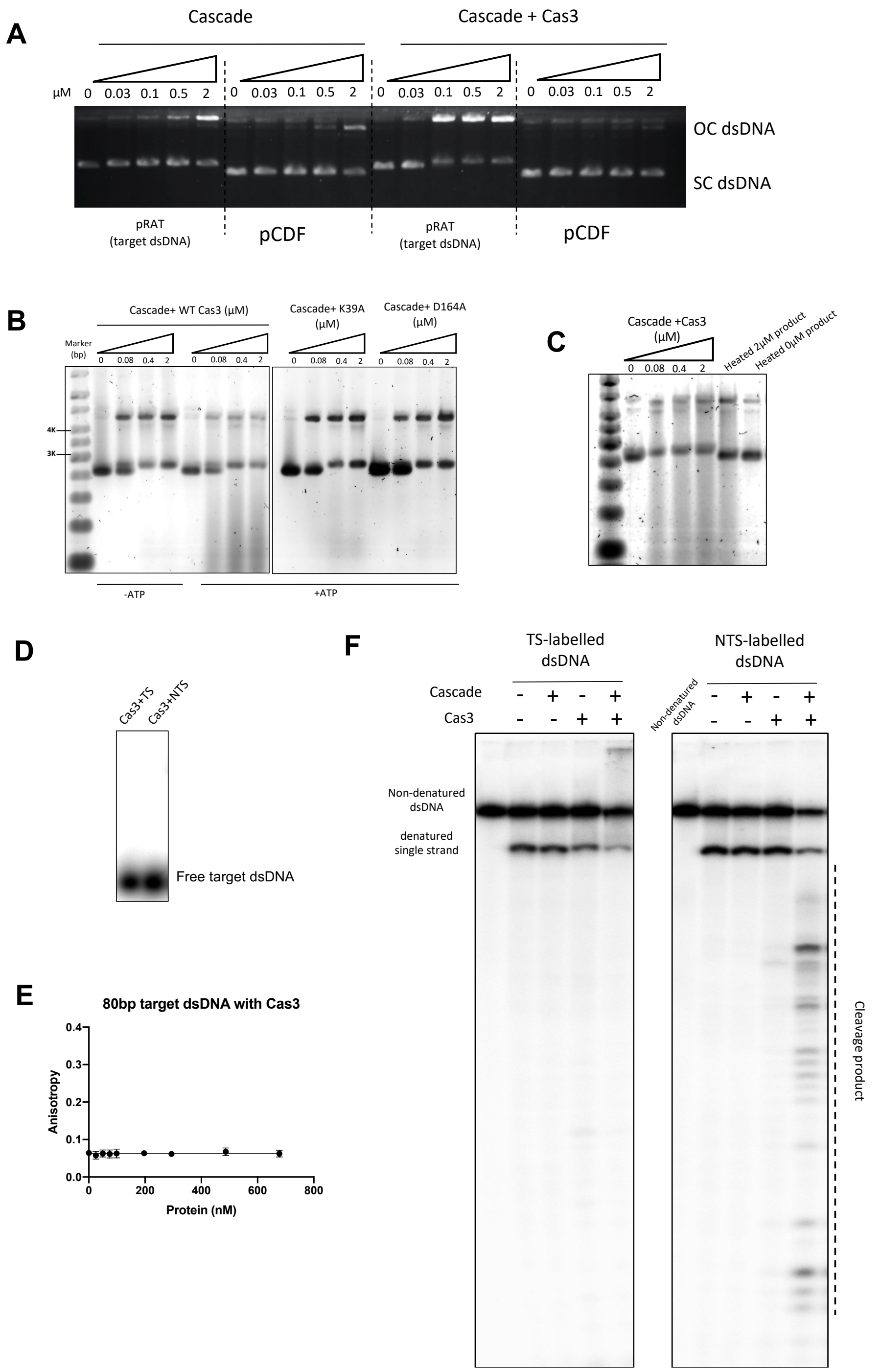

Figure S4. dsDNA targeting and degradation by effector complex. (A) Target dsDNA pRAT and non-target dsDNA pCDF were incubated with type I-G complex cascade or cascade plus Cas3, following an overnight agarose gel electrophoresis. OC (open circular) dsDNA, SC (supercoiled) dsDNA. **(B)** Agarose gel electrophoresis shows that Cas3 helicase domain mutant, Cas3 K39A and D164A aborted the target plasmid degradation. **(C)** Agarose gel electrophoresis shows that shifted band disappeared after heat denaturation. **(D)** Electrophoretic Mobility Shift Assay (EMSA) shows that Cas3 alone has no binding affinity for dsDNA; TS, target strand labelled dsDNA; NTS, non-target strand labelled dsDNA. **(E)** Anisotropy shows that Cas3 alone has no binding affinity for target dsDNA. Data points and error bars represent the mean of five technical replicates and standard deviation. **(F)** Target labelled or NTS-labelled dsDNA was incubated with Cascade and Cas3 in the presence of ATP, products separated on a denaturing polyacrylamide-TBE gel.

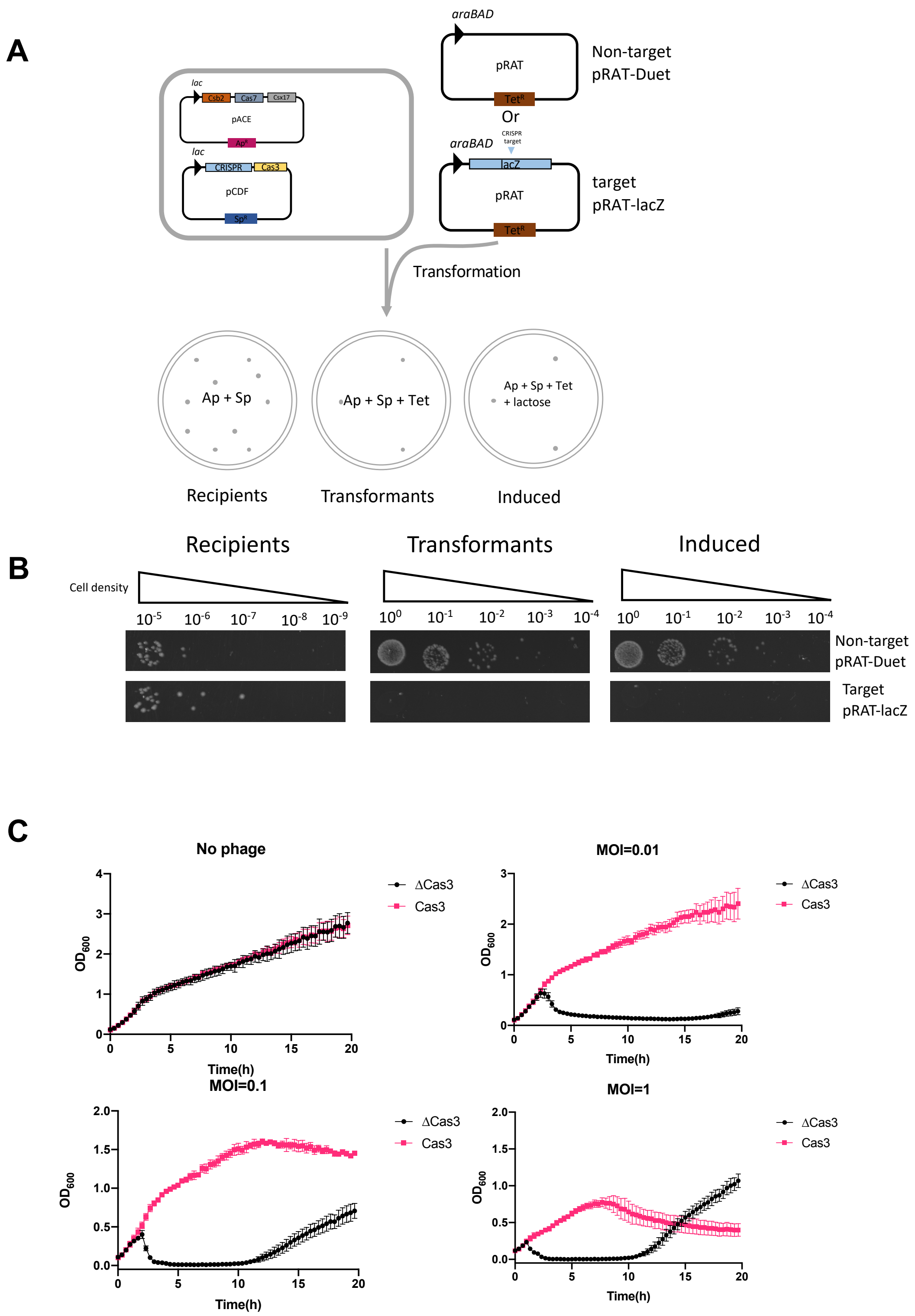

Figure S5. In vivo reconstruction of type I-G. (A) A schematic diagram showing the plasmid challenge assay for target or non-target plasmid. Csb2, Cas7 and Cas8g gene were built in pACE vector, Ampicillin resistenece (Ap); Cas3 gene and CRISPR array targeting the pRAT-lacZ were constructed in pCDF, Spectinomycin resistance (Sp); Blue triangle indicated the CRISPR array target. Recipients, Ampicillin and Spectinomycin in plates; Transformants, Ampicillin, Spectinomycin and tetracycline in plates; Induced, with all three antibiotics and lactose for induction. **(B)** The cells on the plates in different condition. **(C)** Cell growth curve without lactose and arabinose induction. No phage infection or phage infection (MOI=0.01, 0.1 and 1). ΔCas3, cells lack of Cas3 gene. Data points represent the mean of six experimental replicates (two biological replicates and three technical replicates) with standard deviation shown.

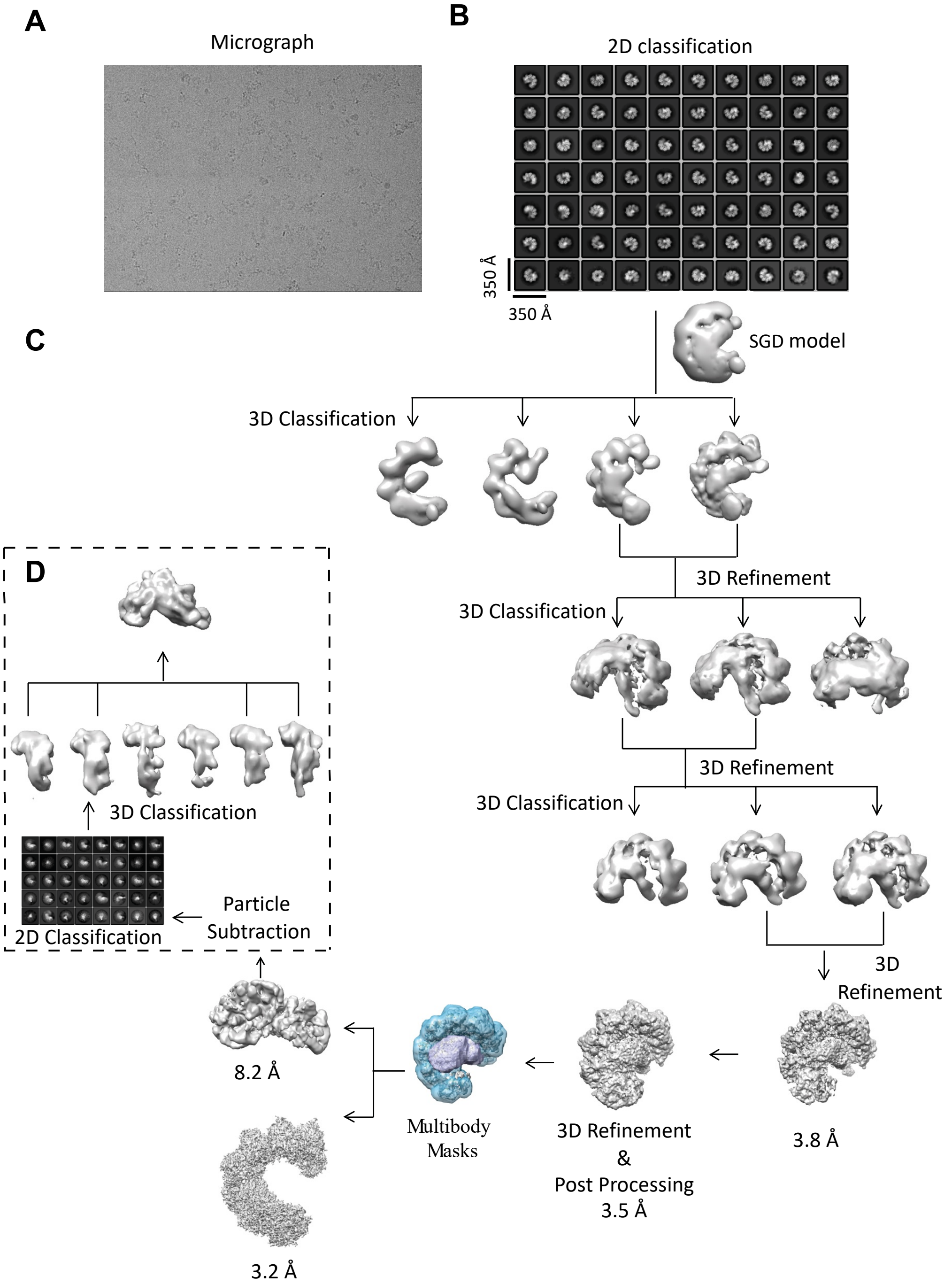

Figure S6. Data processing workflow for type I-G cascade reconstruction. (A) Representative micrograph of vitrified Type IG cascade complex collected in a Titan Krios microscope equipped with Gatan K3 detector. **(B)** Reference free 2D class averages showing various projection images of Type IG cascade present within the dataset. A subset of these particles was used for generation of Initial model using the SGD algorithm implemented in RELION 4. **(C)** Detailed workflow used to identify homogenous set of Type IG cascade complex which is refined to 3.5Å resolution. In each step a hierarchical 3D classification was carried out. The resultant volume is then inspected in chimera and the particles from those volumes with similar features are merged, re-extracted and a 3D refinement was carried out. At the multibody refinement step a soft mask enclosing the Cas7-crRNA array (blue) and the large subunit CSX17 (pale green) was defined. The mask enclosed bodies are then treated as separate bodies and multibody refinement implemented in RELION 4 was carried out. The final Cas7-crRNA volume was refined to 3.2Å and the volume correspond to CSX17 was refined to 8.2Å. **(D)** Isolated particle density through particle subtraction was carried out and centred on the box (200Å) for large subunit CSX17. Subsequently, 2D classification followed by 3D classification was carried out. 3D volumes with similar shapes are merged and a 3D auto refinement was carried.

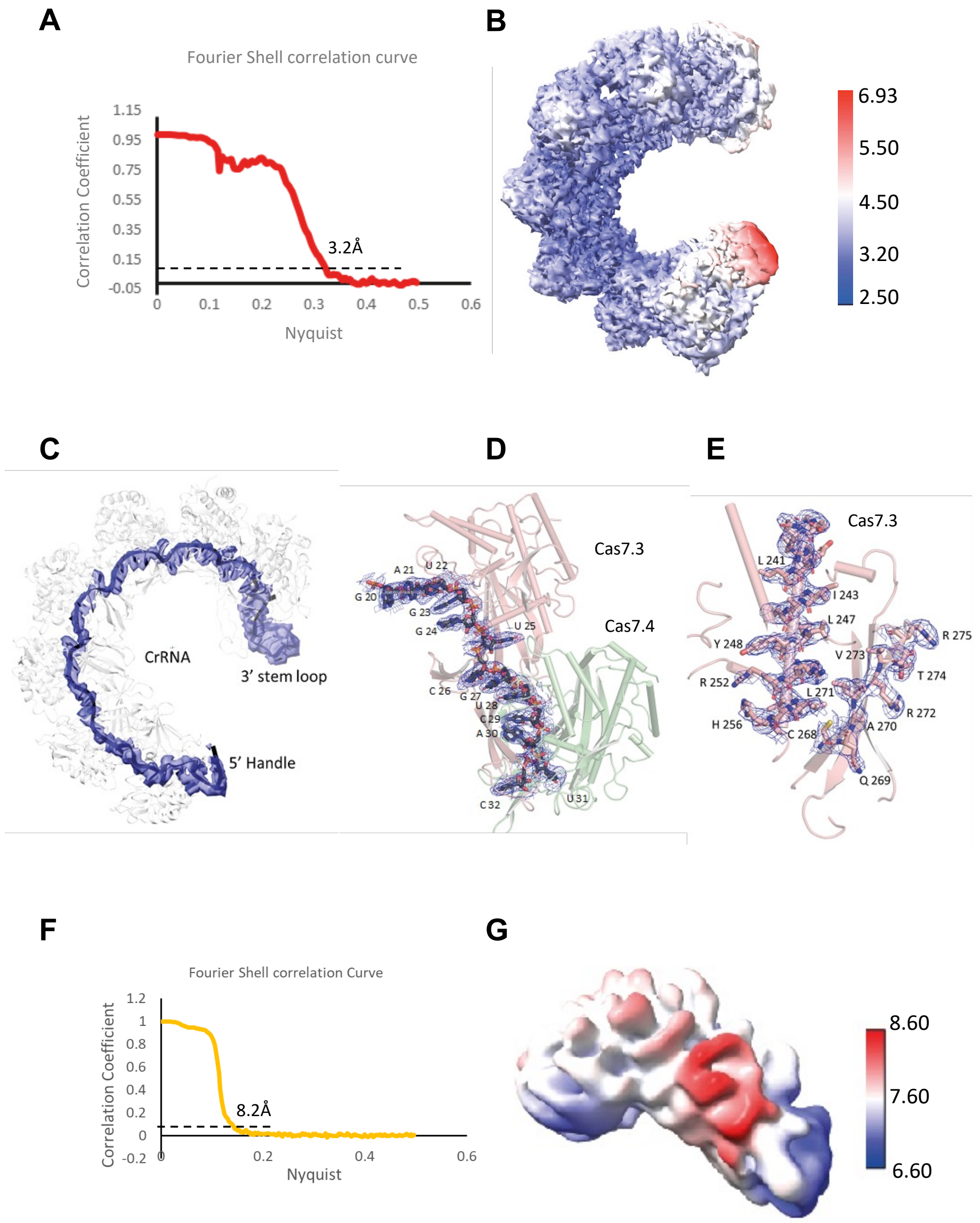

Figure S7. Overall quality of reconstructed Type I-G cascade complex. (A) Gold standard Fourier Shell Correlation curve using half maps. The estimated overall resolution for the reconstruction is 3.2A by the FSC 0.143 criterion. **(B)** Local resolution estimation for the Cas7-crRNA core using RESMAP. The Cas7-crRNA core is shown in surface representation and the surface coloured according to the estimated local resolution value. **(C)** CryoEM density for the crRNA is drawn in surface representation with the refined crRNA shown in cartoon representation. **(D)** A closer view of interaction of crRNA segment with the selected Cas7 subunits. The Cas7 subunits are shown in cartoon representation and the crRNA drawn as stick. The CryoEM density for the crRNA segment is shown in mesh. **(E)** Selected regions of Cas7 residues side chains are shown in stick and the corresponding CryoEM density drawn in mesh. Residues are numbered. **(F)** Gold standard Fourier Shell correlation curve for the Cas8g region. **(G)** Local resolution estimation for the Cas8g volume using RESMAP.

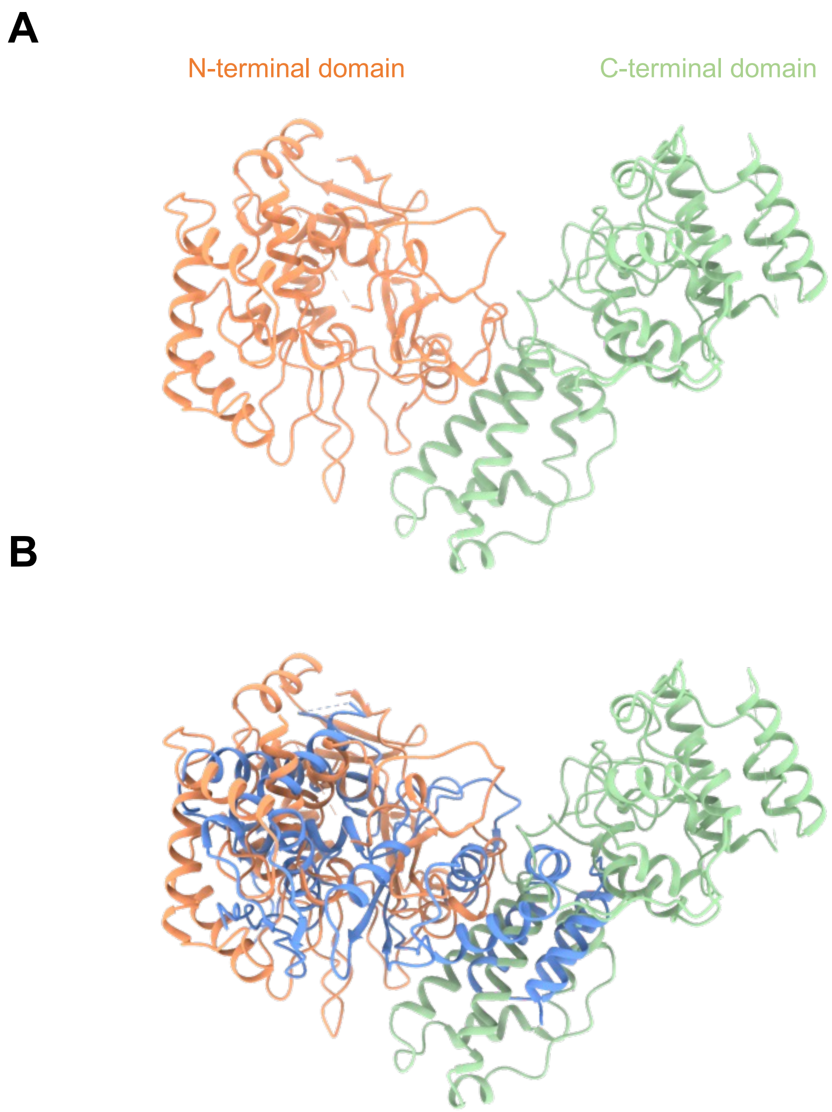

Figure S8. AF2 model of Cas8g/Csx17. (A). The N-terminal domain (orange) has a mixed $\alpha + \beta$ secondary structure while the C-terminal domain (green) is predicted for an α helical bundle, similar to the composition of the Cas11 subunit in other effector complexes. **(B)** structural overlay with the AF2 model of Cas8a2 (blue) from *Methanocaldococcus jannaschii*.

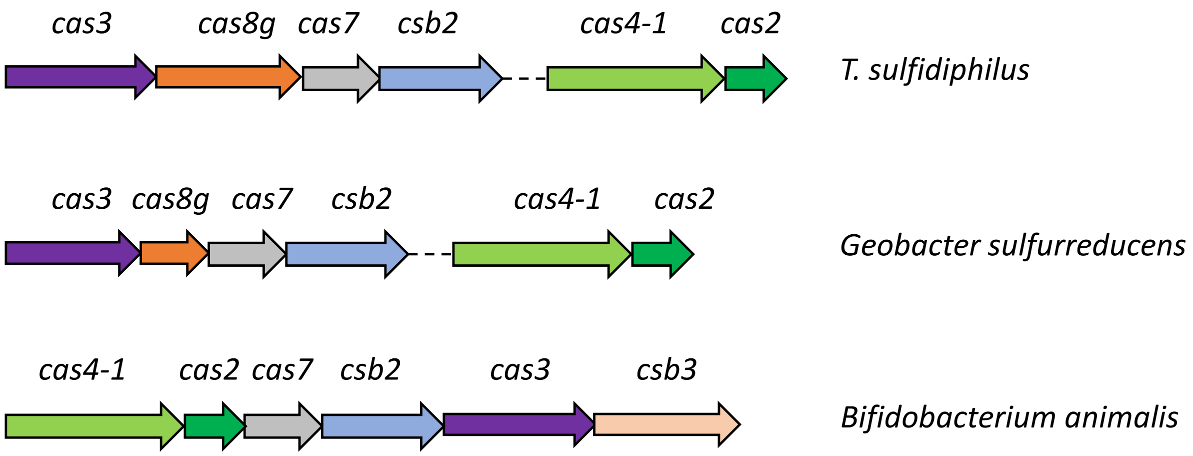

Figure S9. type I-G variants. Adapted from Makarova *et al* (2011). Type I-G variants show variable large subunit Cas8g. Csb3 presumably is the large subunit of Cascade in *Bifidobacterium animalis*.

Supplementary table 1.1 CRISPR array

Name	Squence (5’ to 3’)	Note
TetTarget	GTCATCCGCGGCATTTAGCCGCGGCCTCATTGAAGCAAGCTGTCCCTGATGGTCGTCATCT ACCTGCCTGGAGTCATCCGCGGCATTTAGCCGCGGCCTCATTGAAGCAAGCTGTCCCTGAT GGTCGTCATCTACCTGCCTGGAGTCATCCGCGGCATTTAGCCGCGGCCTCATTGAAGCAAG CTGTCCCTGATGGTCGTCATCTACCTGCCTGGAGTCATCCGCGGCATTTAGCCGCGGCCTC ATTGAAGCAAGCTGTCCCTGATGGTCGTCATCTACCTGCCTGGAGTCATCCGCGGCATTTA GCCGCGGCCTCATTGAAGCAAGCTGTCCCTGATGGTCGTCATCTACCTGCCTGGAGTCATC CGCGGCATTTAGCCGCGGCCTCATTGAAGCAAGCTGTCCCTGATGGTCGTCATCTACCTGC CTGGAGTCATCCGCGGCATTTAGCCGCGGCCTCATTGAAGC	CRISPR array spacer targeting Tetracycline
lacZTarget	GTCATCCGCGGCATTTAGCCGCGGCCTCATTGAAGCCAGCACATCCCCCTTTCGCCAGCTG GCGTAATAGCGGTCATCCGCGGCATTTAGCCGCGGCCTCATTGAAGCCAGCACATCCCCCT TTCGCCAGCTGGCGTAATAGCGGTCATCCGCGGCATTTAGCCGCGGCCTCATTGAAGCCAG CACATCCCCCTTTCGCCAGCTGGCGTAATAGCGGTCATCCGCGGCATTTAGCCGCGGCCTC ATTGAAGCCAGCACATCCCCCTTTCGCCAGCTGGCGTAATAGCGGTCATCCGCGGCATTTA GCCGCGGCCTCATTGAAGC	CRISPR array spacer targeting *lacZ*
lpaTarget	GTCATCCGCGGCATTTAGCCGCGGCCTCATTGAAGCATAGATGACAGTACACGCCCACGTA GATTTCAGATCGTCATCCGCGGCATTTAGCCGCGGCCTCATTGAAGCATAGATGACAGTAC ACGCCCACGTAGATTTCAGATCGTCATCCGCGGCATTTAGCCGCGGCCTCATTGAAGCATA GATGACAGTACACGCCCACGTAGATTTCAGATCGTCATCCGCGGCATTTAGCCGCGGCCTC ATTGAAGC	CRISPR array spacer targeting phage *lpa*

Supplementary table 1.2. Primers

Name	Squence (5’ to 3’)	Note
Csb2 R260 to stop codon	5’- cgttcgctagcgccgtgtaccagagtgg -3’<br>5’- cggcgctagcgaacgcccaacgcaccttg -3;	Primers for mutagenesis
C-terminus of Csb2 amplification	5’- **GCTGCCATGGCT**CATATGTTAGCAGTGGCTTGTCG -3’<br>5’- **CAGCCTCGAGGGATCCTCA**GCGAACGCCCAACGCACC -3’	Primers with NcoI and BamHI site for Csb2 C-terminal domain amplification

Supplementary table 1.3. oligonucleotides

Name	Squence (5’ to 3’)	Note
crRNA repeat	5’-6-FAM- GUCAUCCGCGGCAUUUAGCCGCGGCCUCAUUGAAGC-3’	crRNA repeat with FAM label
3’- Haipin	5’-6-FAM- GUCAUCCGCGGCAUUUAGCCGCGGCCUC-3’	3’haipin with FAM label for anisotropy
5’-8nt-handle	5’- AUUGAAGC-6-FAM- -3’<br>5’-6-FAM- AUUGAAGC-3’	5’-8nt handle for anisotropy, 5’FAM label or 3’FAM label
81nt target strand	5’GCCCGCGTTGCAGGCCATGCTGTCCAGGCAGGTAGATGACGACCATCAG GGACAGCTTCAAGGATCGCTCGCGGCTCTTAC-3’	Tetracycline resistance target
80nt non-target strand	5’GTAAGAGCCGCGAGCGATCCTTGAAGCTGTCCCTGATGGTCGTCATCTAC CTGCCTGGACAGCATGGCCTGCAACGCGGG -3’	Complementary strand for dsDNA duplex

Supplementary table 2.

**Data collection**	
Magnification	105,000
Voltage (kV)	300
Electron exposure (e-/Å2)	40
Frames in movies (no.)	36
Defocus range (µm)	1.5-3.2
Pixel size (Å)	0.84
**Reconstruction**	
Symmetry imposed	C1
Initial particle images (no.)	3,420,096
Final particle images (no.)	415,000
Map resolution (Å)	3.8
FSC threshold	0.143
Map resolution range (Å)	3.8-8
Sharpening B-factor (Å2)	-188
**Refinement and Validation**	
Subunits	Cas7, Csx17, Nucleotide
Model composition	
Number of Chains	8
Protein residues	2212
RNA(no.bases)	66
Non-hydrogen atoms	36740
Hydrogen atoms	18195
**Root mean square deviation**	
Bond lengths (Å)	0.008
Bond Angles (Å)	1.025
MolProbity Score	1.23
Clashscore	3.6
Poor rotamers	0.31
Ramachandran Plot	
Favoured (%)	95.2
Allowed (%)	4.8
