# Supplementary material for: Structure and mechanism of the type I-G CRISPR effector
Source: Nucleic Acids Res. 2022 Oct 28;50(19):11214–28. doi: 10.1093/nar/gkac925 (PMC9638904; doi:10.1093/nar/gkac925)
Supplement: gkac925_Supplemental_Files [file gkac925_supplemental_files.zip › Supplementary Movie 1 flgure legend.pdf]

Supplementary Movie 1. Comparison of crRNA structures from type I CRISPR effectors, aligned at the 5’ end of the crRNA.
